# Data on individual metabolites of synthetic cannabinoids JWH-018, JWH-073 and AM2201 by *Cunninghamella elegans*

**DOI:** 10.1016/j.dib.2016.02.039

**Published:** 2016-02-23

**Authors:** Shimpei Watanabe, Unnikrishnan Kuzhiumparambil, Zophia Winiarski, Shanlin Fu

**Affiliations:** a Centre for Forensic Science, School of Mathematical and Physical Sciences, University of Technology Sydney (UTS), Australia; b Plant Functional Biology and Climate Change Cluster, University of Technology Sydney (UTS), Australia; c Cell Biology Facility, University of Technology Sydney (UTS), Australia

## Abstract

Synthetic cannabinoids JWH-018, JWH-073 and AM2201 were metabolised by the fungus *Cunninghamella elegans*. In this article, data on individual metabolites of their retention times, mass accuracies, major product ions and structures indicated by product ions are presented. The data in this article is related to “Biotransformation of synthetic cannabinoids JWH-018, JWH-073 and AM2201 by *Cunninghamella elegans*” [1].

**Specifications Table**

**Table:** 

Subject area	Pharmacology
More specific subject area	Drug Metabolism
Type of data	Table
How data was acquired	Liquid chromatography -tandem mass spectrometry (Agilent 1290 LC system coupled to Agilent 6490 Triple Quadrupole mass spectrometer), high resolution quadrupole Time-of-Flight mass spectrometry (Agilent 6510 Accurate Mass QToF Mass Spectrometer)
Data format	Analysed
Experimental factors	Samples were extracted by dichloromethane.
Experimental features	Liquid chromatography-tandem mass spectrometry analysis of fungal metabolites of synthetic cannabinoids JWH-018, JWH-073 and AM2201
Data source location	Sydney, Australia
Data accessibility	Data are available with this article

**Value of the data**

• Chromatographic and mass spectrometric data on individual metabolites are provided for reference.
• Product ions indicative of the structures of metabolites are listed.
• The data can be compared with human or other in vitro metabolism.

## 1 Data

Table 1, Table 3, Table 5 list all the metabolites with biotransformation, retention time, observed accurate mass, formula and major product ions of JWH-018, JWH-073 and AM2201, respectively. Product ions representative of structures for JWH-018, JWH-073 and AM2201 metabolites are presented in Table 4, Table 6, respectively. Overlaid extracted ion chromatograms of all the metabolites of JWH-018, JWH-073 and AM2201 are shown in Fig. 2 of Ref. [1] with annotated metabolite identification (ID) names (Table 1, Table 2, Table 3, Table 4, Table 5, Table 6).

## 2 Experimental design, materials and methods

### 2.1 Chemicals

JWH-018 and JWH-073 were synthesized in-house following previously reported methods and characterized by mass spectrometry (MS) and 1D, 2D nuclear magnetic resonance (NMR) spectroscopy [3], [4]. AM2201 (purity 99.4%) was obtained from the National Measurement Institute (North Ryde, NSW, Australia). Reference standards JWH-018 *N*-(4-hydroxypentyl), JWH-018 *N*-(5-hydroxypentyl), JWH-018 *N*-pentanoic acid, JWH-073 *N*-(3-hydroxybutyl), JWH-073 *N*-(4-hydroxybutyl) and JWH-073 *N*-butanoic acid were obtained from PM separations (Capalaba, QLD, Australia). Reagent grade dichloromethane, methanol, KH_2_PO_4_, NaCl and LC grade acetonitrile and methanol were obtained from Chemsupply (Gilman, SA, Australia). Potato dextrose agar, glucose, peptone, and yeast extract were purchased from Oxoid Australia (Adelaide, SA, Australia).

### 2.2 Microbial culture and biotransformation conditions

Cultures of *C. elegans* ATCC 10028b (Cryosite Ltd, South Granville, NSW, Australia) were propagated on potato dextrose agar plates at 27 °C for 5 days. The mycelia from five plates were then transferred to 20 mL of sterile physiological saline solution and homogenized for 5 min. Approximately 3 mL aliquots of the homogenate were used to inoculate 250 mL Erlenmeyer flasks containing 100 ml of growth media. The cultures were incubated for 48 h at 26 °C on an Infors HT Multitron rotary shaker (*in vitro* Technologies, Noble Park North, VIC, Australia) operating at 180 rpm. After 48 h, 10 mg of JWH-018, JWH-073 or AM2201 dissolved in 0.5 mL of methanol was added to the culture and incubated for further 72 h [5]. Control experiments consisted of cultures without cannabinoids and flasks containing only media and cannabinoid [6], [7].

### 2.3 Extraction, isolation, and identification of metabolites

After 72 h of incubation, the contents of each flask, including the controls, were filtered through Buchner funnel into a separating funnel and extracted with three aliquots of dichloromethane (3×50 mL). The combined organic extracts were evaporated to dryness under vacuum at 40 °C using a Buchi rotary evaporator (*in vitro* Technologies, Noble Park North, VIC, Australia) and placed under high vacuum to remove traces of moisture. The residue was dissolved in acetonitrile to prepare 1 mg/mL stock solution and was filtered through 0.22 µM syringe filter before analysis. Cannabinoid parent drugs and metabolites were chromatographically separated using an Agilent Zorbax Eclipse XDB-C18 analytical column (150×4.6 mm, 5 μm). Mobile phases consisted of 0.1% formic acid in water (mobile phase A) and acetonitrile (mobile phase B). The gradient used consisted of 30% B (0 to 2 min), linear gradient from 30% B to 50% B (2 to 5 min), 50% B to 90% B (5 to 30 min, hold for 5 min) and 90% B to 30% B (35 to 40 min) run at 0.4 mL/min. MS data were acquired on an Agilent 6490 Triple Quadrupole mass spectrometer with an electrospray ionization source (ESI) source (positive ion mode), interfaced with an Agilent 1290 LC system. Samples prepared were injected in 2 µL volume to obtain full scan and product ion scan spectra. Product ion scan experiments were conducted on precursor ions that were presumed to be metabolites based on the comparison of full scan spectra of the samples and controls. A fragmentor voltage of 380 V with discrete collision energy of 10, 20, 30 and 40 eV (for product ion scan) was applied. The scanning mass range was set at *m*/*z* 100–1000 (scan time=500 ms). The sheath gas temperature and flow were set to 250 °C and 11 L/min, respectively. The capillary and nozzle voltages were 3000 V and 1500 V, respectively.

High resolution quadrupole Time-of-Flight mass spectrometry (HRQToFMS) experiments were carried out on an Agilent 6510 Accurate Mass QToF Mass Spectrometer, equipped with ESI source operated in positive ion mode, in order to determine accurate masses of the metabolites. The LC system and conditions used were the same as above. The following operation parameters were used: injection volume 2 µL (full scan) and 10 µL (product ion scan); capillary voltage 3500 V; nebulizer pressure 40 psi (275790 Pa); drying gas 10.0 L/min; gas temperature 350 °C; fragmentor voltage 160 V; collision energy 10, 20 and 40 eV; skimmer voltage 60 V. HRQToFMS accurate mass spectra were recorded across the range from *m*/*z* 100 to *m*/*z* 1000. The mass axis was calibrated using the mixture provided by the manufacturer over the *m*/*z* 50–3200 range. A second orthogonal sprayer with a reference solution was used as a continuous calibration using the following reference masses: *m*/*z* 121.0509 and *m*/*z* 922.0098. The chromatographic conditions and column used were same as described above. The controls were subjected to the same analysis. Analysis of the chromatographic and mass spectrometric data was conducted using MassHunter Workstation Software Qualitative Analysis (version B.06.00, Agilent). Peaks present in the fungus sample, but not in the controls, were manually identified and their fragmentation patterns and accurate masses were examined to identify the metabolites. The signal-to-noise ratio of all the identified metabolites was greater than 5.

## Figures and Tables

**Table 1:** Metabolites of JWH-018 after *C. elegans* incubation.

ID	Biotransformation	RT, min	*m*/*z* [M+H]^+^	Mass accuracy (ppm)	Formula	Major product ions
Ma1	Dihydrodiol formation+*N*-dealkylation	8.0	306.1123	−0.7	C_19_H_15_NO_3_	143, 144, 171, 189
Ma2	Dihydrodiol formation+hydroxylation at pentyl side chain	8.5	392.1852	−1.0	C_24_H_25_NO_4_	143, 144, 171, 189, 230, 374
Ma3	Dihydrodiol formation+hydroxylation at pentyl side chain	9.2	392.1850	−1.6	C_24_H_25_NO_4_	143, 144, 171, 189, 230, 374
Ma4	Dihydrodiol formation+ketone formation at pentyl side chain	9.3	390.1694	−1.4	C_24_H_23_NO_4_	143, 144, 171, 189, 228
Ma5	Dihydrodiol formation+ketone formation at pentyl side chain	10.3	390.1695	−1.1	C_24_H_23_NO_4_	143, 144, 171, 189, 228
Ma6	Dihydroxylation at pentyl side chain and naphthalene moiety	12.5	374.1745	−1.5	C_24_H_23_NO_3_	143, 144, 171, 230
Ma7	Dihydroxylation at pentyl side chain	12.8	374.1745	−1.6	C_24_H_23_NO_3_	127, 144, 155, 246
Ma8	Dihydroxylation at pentyl side chain and naphthalene moiety	13.1	374.1746	−1.3	C_24_H_23_NO_3_	143, 144, 171, 230
Ma9	Dihydroxylation at pentyl side chain and naphthalene moiety	14.1	374.1746	−1.3	C_24_H_23_NO_3_	143, 144, 171, 230
Ma10	Ketone formation at pentyl side chain+hydroxylation at naphthalene moiety	14.5	372.1590	−1.0	C_24_H_21_NO_3_	143, 144, 171, 228
Ma11	Dihydroxylation at pentyl side chain and naphthalene moiety	14.9	374.1745	−1.5	C_24_H_23_NO_3_	143, 144, 171, 230
Ma12	Dihydrodiol formation	16.1	376.1901	−1.6	C_24_H_25_NO_3_	143, 171, 189, 214
Ma13	Carboxylation (*N*-pentanoic acid)^a^	16.4	372.1591	−0.9	C_24_H_21_NO_3_	127, 144 155 244
Ma14	Hydroxylation at pentyl side chain (5-hydroxypentyl)^a^	17.2	358.1796	−1.6	C_24_H_23_NO_2_	127, 144, 155, 230
Ma15	Hydroxylation at pentyl side chain (4-hydroxypentyl)^a^	17.6	358.1798	−1.0	C_24_H_23_NO_2_	127, 144, 155, 230
Ma16	Hydroxylation at pentyl side chain	19.9	358.1798	−1.1	C_24_H_23_NO_2_	127, 144, 155, 230
Ma17	Ketone formation at pentyl side chain	20.2	356.1640	−1.6	C_24_H_21_NO_2_	127, 144, 155, 228
Ma18	Ketone formation at pentyl side chain	22.4	356.1641	−1.2	C_24_H_21_NO_2_	127, 144, 155, 228
Ma19	Hydroxylation at naphthalene moiety	24.3	358.1797	−1.3	C_24_H_23_NO_2_	143, 171, 214
Ma20	Hydroxylation at naphthalene moiety	24.8	358.1797	−1.2	C_24_H_23_NO_2_	143, 171, 214
Ma21	Dehydrogenation	28.7	340.1692	−1.1	C_24_H_21_NO	127, 144, 155, 212
	JWH-018	31.4	342.1851	−0.5	C_24_H_23_NO	127, 144, 155, 214

a Position confirmed by the use of reference standards.

**Table 2:** Key diagnostic product ions and their tentative structures used in elucidating biotransformation pathways of JWH-018 after *C. elegans* incubation.

Biotransformation	ID	Key diagnostic product ions (*m*/*z)* and tentative structures
Carboxylation at pentyl side chain	Ma13	144: unchanged indole, 244: carboxylated pentylindole
Dehydrogenation at pentyl side chain	Ma21	144: unchanged indole, 212: dehydrogenated *N*-pentylindole
Dihydrodiol formation at naphthalene moiety	Ma12	189: naphthalene with dihydrodiol
Dihydrodiol formation at naphthalene moiety+hydroxylation at pentyl side chain	Ma2, Ma3	144: unchanged indole, 189: naphthalene with dihydrodiol, 230: hydroxylated *N*-pentylindole
Dihydrodiol formation at naphthalene moiety+ketone formation at pentyl side chain	Ma4, Ma5	189: naphthalene with dihydrodiol, 228: *N*-pentylindole with ketone
Dihydrodiol formation at naphthalene moiety+*N*-dealkylation	Ma1	189: naphthalene with dihydrodiol
Dihydroxylation at pentyl side chain	Ma7	144: unchanged indole, 246: dihydroxylated *N*-pentylindole
Dihydroxylation at pentyl side chain and naphthalene moiety	Ma6, Ma8, Ma9, Ma11	144: unchanged indole, 171: hydroxylated naphthalene, 230: hydroxylated *N*-pentylindole
Hydroxylation at naphthalene moiety	Ma19, Ma20	171: hydroxylated naphthalene
Hydroxylation at pentyl side chain	Ma14 – Ma16	144: unchanged indole, 230: hydroxylated *N*-pentylindole
Ketone formation at pentyl side chain	Ma17, Ma18	144: unchanged indole, 228: *N*-pentylindole with ketone
Ketone formation at pentyl side chain+hydroxylation at naphthalene moiety	Ma10	144: unchanged indole, 171: hydroxylated naphthalene, 228: *N*-pentylindole with ketone

**Table 3:** Metabolites of JWH-073 after *C. elegans* incubation.

ID	Biotransformation	RT, min	*m*/*z* [M+H]^+^	Mass accuracy (ppm)	Formula	Major product ions
Mb1	Dihydrodiol formation+*N*-dealkylation	8.0	306.1123	−0.5	C_19_H_15_NO_3_	143, 144, 171
Mb2	Dihydrodiol formation+hydroxylation at butyl side chain	8.2	378.1700	−0.1	C_23_H_23_NO_4_	143, 144, 171, 189, 216
Mb3	Dihydrodiol formation+hydroxylation at butyl side chain	8.9	378.1690	−2.6	C_23_H_23_NO_4_	143, 144, 171, 189, 216
Mb4	Dihydrodiol formation+ketone formation at butyl side chain	9.0	376.1543	−0.1	C_23_H_21_NO_4_	143, 144, 171, 189, 214
Mb5	Dihydroxylation at butyl side chain	11.7	360.1594	0.0	C_23_H_21_NO_3_	127, 144, 155, 232
Mb6	Dihydroxylation at butyl side chain and naphthalene moiety	12.1	360.1595	0.1	C_23_H_21_NO_3_	143, 144, 171, 216
Mb7	Dihydroxylation at butyl side chain and naphthalene moiety	12.8	360.1594	−0.1	C_23_H_21_NO_3_	143, 144, 171, 216
Mb8	Dihydrodiol formation	13.7	362.1751	0.1	C_23_H_23_NO_3_	143, 144, 171, 189, 200
Mb9	Ketone formation at butyl side chain+hydroxylation at naphthalene moiety	13.9	358.1438	0.0	C_23_H_19_NO_3_	143, 144, 171, 214
Mb10	Ketone formation at butyl side chain+hydroxylation at naphthalene moiety	14.5	358.1438	0.0	C_23_H_19_NO_3_	143, 144, 171, 214
Mb11	Carboxylation (*N*-butanoic acid)^a^	15.4	358.1437	−0.1	C_23_H_19_NO_3_	127, 144, 155, 230
Mb12	Hydroxylation at butyl side chain (4-hydroxybutyl)^a^	15.7	344.1645	−0.2	C_23_H_21_NO_2_	127, 144, 155, 216
Mb13	Hydroxylation at butyl side chain (3-hydroxybutyl)^a^	17.1	344.1646	0.3	C_23_H_21_NO_2_	127, 144, 155, 216
Mb14	Ketone formation at butyl side chain	19.5	342.1488	0.0	C_23_H_19_NO_2_	127, 144, 155, 214
Mb15	Hydroxylation at naphthalene moiety	21.5	344.1645	−0.1	C_23_H_21_NO_2_	143, 144, 171, 200
Mb16	Hydroxylation at naphthalene moiety	22.0	344.1645	0.0	C_23_H_21_NO_2_	143, 144, 171, 200
Mb17	Dehydrogenation	25.7	326.1539	−0.1	C_23_H_19_NO	127, 155, 198
	JWH-073	28.8	328.1697	0.3	C_23_H_21_NO	127, 144, 155, 200

a Position confirmed by the use of reference standards.

**Table 4:** Key diagnostic product ions and their tentative structures used in elucidating biotransformation pathways of JWH-073 after *C. elegans* incubation.

Biotransformation	ID	Key diagnostic product ions (*m*/*z)* and tentative structures
Carboxylation at butyl side chain	Mb11	144: unchanged indole, 230: carboxylated butylindole
Dehydrogenation	Mb17	198: dehydrogenated *N*-butylindole
Dihydrodiol formation at naphthalene moiety	Mb8	189: naphthalene with dihydrodiol
Dihydrodiol formation at naphthalene moiety+hydroxylation at butyl side chain	Mb2, Mb3	144: unchanged indole, 189: naphthalene with dihydrodiol, 216: hydroxylated *N*-butylindole
Dihydrodiol formation at naphthalene moiety+ketone formation at butyl side chain	Mb4	144: unchanged indole, 189: naphthalene with dihydrodiol, 214: *N*-butylindole with ketone
Dihydrodiol formation at naphthalene moiety+*N*-dealkylation	Mb1	171: hydroxylated naphthalene (resulting from naphthalene with dihydrodiol [2])
Dihydroxylation at butyl side chain	Mb5	144: unchanged indole, 232: dihydroxylated *N*-butylindole
Dihydroxylation at butyl chain and naphthalene moiety	M6, Mb7	144: unchanged indole, 171: hydroxylated naphthalene, 216: hydroxylated *N*-butylindole
Hydroxylation at butyl side chain	Mb12, Mb13	144: unchanged indole, 216: hydroxylated *N*-butylindole
Hydroxylation at naphthalene moiety	Mb15, Mb16	171: hydroxylated naphthalene
Ketone formation at butyl side chain	Mb14	144: unchanged indole, 214: *N*-butylindole with ketone
Ketone formation at butyl side chain+hydroxylation at naphthalene moiety	Mb9, Mb10	144: unchanged indole, 171: hydroxylated naphthalene, 214: *N*-butylindole with ketone

**Table 5:** Metabolites of AM2201 after *C. elegans* incubation.

ID	Biotransformation	RT, min	*m*/*z* [M+H]^+^	Mass accuracy (ppm)	Formula	Major product ions
Mc1	Dihydrodiol formation+*N*-dealkylation	8.0	306.1122	−1.0	C_19_H_15_NO_3_	143, 144, 171, 189
Mc2	Dihydroxylation at pentyl side chain and naphthalene moiety+glucosidation	8.2	554.2185	0.0	C_30_H_32_FNO_8_	143, 144, 171, 248, 392
Mc3	Dihydroxylation at pentyl side chain and naphthalene moiety+glucosidation	8.4	554.2186	0.2	C_30_H_32_FNO_8_	143, 144, 171, 248, 392
Mc4	Oxidative defluorination+dihydrodiol formation (JWH-018 dihydrodiol-hydroxy)	8.5	392.1858	0.4	C_24_H_25_NO_4_	143, 171, 189, 230
Mc5	Dihydrodiol formation+hydroxylation at pentyl side chain	8.5	410.1764	0.6	C_24_H_24_FNO_4_	143, 144, 171, 189, 248
Mc6	Dihydrodiol formation+hydroxylation at pentyl side chain	8.7	410.1761	−0.2	C_24_H_24_FNO_4_	143, 144, 171, 189, 248
Mc7	Dihydroxylation at pentyl side chain and/or indole moiety+glucosidation	9.0	554.2186	0.3	C_30_H_32_FNO_8_	127, 155, 264, 392
Mc8	Trihydroxylation at pentyl side chain, indole moiety and naphthalene moiety	9.3	408.1606	0.1	C_24_H_22_FNO_4_	143, 160, 171, 264
Mc9	Dihydrodiol formation+dihydroxylation at indole moiety	9.5	426.1712	0.1	C_24_H_24_FNO_5_	143, 171, 176, 189, 264
Mc10	Dihydrodiol formation+ketone formation at pentyl side chain	9.7	408.1606	0.0	C_24_H_22_FNO_4_	143, 171, 189, 246
Mc11	Trihydroxylation at pentyl side chain, indole moiety and naphthalene moiety	10.2	408.1606	0.0	C_24_H_22_FNO_4_	143, 160, 171, 264
Mc12	Hydroxylation at indole moiety+glucosidation	10.5	538.2235	−0.1	C_30_H_32_FNO_7_	127, 155, 160, 248, 376
Mc13	Dihydroxylation at pentyl side chain and/or indole moiety+glucosidation	10.7	554.2183	−0.3	C_30_H_32_FNO_8_	127, 155, 264, 392
Mc14	Hydroxylation at naphthalene moiety+glucosidation	10.8	538.2234	−0.4	C_30_H_32_FNO_7_	143, 144, 171, 232, 376
Mc15	Oxidative defluorination+hydroxylation at indole moiety (JWH-018 dihydroxy)	11.1	374.1751	0.2	C_24_H_23_NO_3_	127, 155, 160, 246
Mc16	Dihydroxylation at naphthalene+glucosidation	11.5	554.2182	−0.4	C_30_H_32_FNO_8_	187, 232, 392
Mc17	Dihydroxylation at indole moiety and pentyl side chain	11.5	392.1657	0.0	C_24_H_22_FNO_3_	127, 155, 160, 264
Mc18	Oxidative defluorination+hydroxylation at indole moiety (JWH-018 dihydroxy)	11.8	374.1748	−0.8	C_24_H_23_NO_3_	127, 155, 160, 246
Mc19	Dihydroxylation at indole moiety and pentyl side chain	11.9	392.1656	−0.2	C_24_H_22_FNO_3_	127, 155, 160, 264
Mc20	Oxidative defluorination to carboxylic acid+hydroxylation at naphthalene moiety	12.0	388.1547	1.0	C_24_H_21_NO_4_	143, 144, 171, 244
Mc21	Oxidative defluorination+hydroxylation at pentyl side chain (JWH-018 dihydroxy)	12.1	374.1751	0.2	C_24_H_23_NO_3_	127, 144, 155, 246
Mc22	Dihydroxylation at indole moiety and pentyl side chain	12.2	392.1658	0.5	C_24_H_22_FNO_3_	127, 155, 160, 264
Mc23	Oxidative defluorination+hydroxylation at naphthalene moiety (JWH-018 dihydroxy)	12.3	374.1749	−0.4	C_24_H_23_NO_3_	143, 144, 171, 230
Mc24	Dihydroxylation at pentyl side chain and naphthalene moiety	12.5	392.1651	1.4	C_24_H_22_FNO_3_	143, 144, 171, 248
Mc25	Dihydrodiol formation	12.5	394.1812	−0.3	C_24_H_24_FNO_3_	143, 144, 171, 189, 232
Mc26	Oxidative defluorination to carboxylic acid+hydroxylation at naphthalene moiety	12.7	388.1544	0.3	C_24_H_21_NO_4_	143, 144, 171, 244
Mc27	Dihydroxylation at indole moiety and pentyl side chain	12.7	392.1657	0.0	C_24_H_22_FNO_3_	127, 155, 160, 264
Mc28	Oxidative defluorination+hydroxylation at naphthalene moiety (JWH-018 dihydroxy)	12.9	374.1750	−0.3	C_24_H_23_NO_3_	143, 144, 171, 230
Mc29	Dihydroxylation at pentyl side chain and naphthalene moiety	13.0	392.1655	0.3	C_24_H_22_FNO_3_	143, 144, 171, 248
Mc30	Dihydrodiol formation	13.5	394.1818	1.2	C_24_H_24_FNO_3_	143, 144, 171, 189, 232
Mc31	Dihydroxylation at pentyl side chain and naphthalene moiety	13.6	392.1655	−0.3	C_24_H_22_FNO_3_	143, 144, 171, 248
Mc32	Dihydroxylation at pentyl side chain and naphthalene moiety	14.4	392.1659	0.6	C_24_H_22_FNO_3_	143, 144, 171, 248
Mc33	Oxidative defluorination to carboxylic acid (JWH-018 *N*-pentanoic acid)^a^	16.4	372.1592	−0.7	C_24_H_21_NO_3_	127, 144, 155, 244
Mc34	Oxidative defluorination (JWH-018 *N*-(5-hydroxypentyl))^a^	17.1	358.1802	0.2	C_24_H_23_NO_2_	127, 144, 155, 230
Mc35	Hydroxylation at pentyl side chain	17.3	376.1709	0.4	C_24_H_22_FNO_2_	127, 144, 155, 248
Mc36	Hydroxylation at indole moiety	17.7	376.1707	−0.2	C_24_H_22_FNO_2_	127, 155, 160, 248
Mc37	Hydroxylation at pentyl side chain	18.0	376.1708	0.1	C_24_H_22_FNO_2_	127, 144, 155, 248
Mc38	Dihydroxylation at naphthalene moiety+sulfation	18.7	472.1222	−0.6	C_24_H_22_FNO_6_S	144, 158, 159, 186, 187, 232, 391, 392
Mc39	Hydroxylation at pentyl side chain	18.8	376.1708	0.3	C_24_H_22_FNO_2_	127, 144, 155, 248
Mc40	Hydroxylation at naphthalene moiety	19.2	376.1709	0.6	C_24_H_22_FNO_2_	143, 144, 171, 232
Mc41	Hydroxylation at naphthalene moiety	19.8	376.1703	−1.2	C_24_H_22_FNO_2_	143, 144, 171, 232
Mc42	Dihydroxylation at naphthalene moiety+sulfation	20.2	472.1224	−0.1	C_24_H_22_FNO_6_S	144, 158, 159, 186, 187, 232, 391, 392
Mc43	Ketone formation at pentyl side chain	20.4	374.1549	−0.5	C_24_H_20_FNO_2_	127, 144, 155, 246
Mc44	Hydroxylation at naphthalene moiety	20.6	376.1706	−0.3	C_24_H_22_FNO_2_	143, 144, 171, 232
Mc45	Hydroxylation at naphthalene moiety	21.3	376.1707	−0.1	C_24_H_22_FNO_2_	143, 144, 171, 232
Mc46	Dihydroxylation at indole moiety+sulfation	22.0	472.1223	−0.3	C_24_H_22_FNO_6_S	127, 155, 175, 176, 264, 391, 392
	AM2201	25.6	360.1759	0.2	C_24_H_22_FNO	127, 144, 155, 232
Mc47	Defluorination+Demethylation (JWH-073)^a^	28.8	328.1695	−0.3	C_23_H_21_NO	127, 144, 155, 200
Mc48	Defluorination (JWH-018)^a^	31.5	342.1853	0.0	C_24_H_23_NO	127, 144, 155, 214

a Position confirmed by the use of reference standards.

**Table 6:** Key diagnostic product ions and their tentative structures used in elucidating biotransformation pathways of AM2201 after *C. elegans* incubation. Square brackets indicate phase II metabolism.

Biotransformation	ID	Key diagnostic product ions (*m/z)* and tentative structures
Defluorination (JWH-018)	Mc48	155: unchanged naphthalene, 214: unchanged *N*-pentylindole
Defluorination+Demethylation (JWH-073)	Mc47	155: unchanged naphthalene, 200: unchanged *N*- butylindole
Dihydrodiol formation at naphthalene moiety	Mc25, Mc30	189: naphthalene with dihydrodiol
Dihydrodiol formation at naphthalene moiety+dihydroxylation at indole moiety	Mc9	176: dihydroxylated indole, 189: naphthalene with dihydrodiol
Dihydrodiol formation at naphthalene moiety+hydroxylation at pentyl side chain	Mc5, Mc6	144: unchanged indole, 189: naphthalene with dihydrodiol, 248: hydroxylated *N*-fluoropentylindole
Dihydrodiol formation at naphthalene moiety+ketone formation at pentyl side chain	Mc10	144: unchanged indole, 189: naphthalene with dihydrodiol, 246: *N*-fluoropentylindole with ketone
Dihydrodiol formation at naphthalene moiety+*N*-dealkylation	Mc1	189: naphthalene with dihydrodiol
Dihydroxylation at indole moiety [+sulfation]	[Sulfate Mc46]	176: dihydroxylation at indole, [392: dihydroxylated AM2201]
Dihydroxylation at indole moiety and pentyl side chain	Mc17, Mc19, Mc22, Mc27	160: hydroxylated indole, 264: dihydroxylated *N*-fluoropentylindole
Dihydroxylation at naphthalene moiety [+glucosidation and sulfation]	[Glucoside Mc16, sulfates Mc38, Mc42]	187: dihydroxylated naphthalene, [392: dihydroxylated AM2201]
Dihydroxylation at naphthalene moiety and pentyl side chain [+glucosidation]	Mc24, Mc29, Mc31, Mc32, [glucosides Mc2, Mc3]	144: unchanged indole, 171: hydroxylated naphthalene, 248: hydroxylated *N*-fluoropentylindole, [392: dihydroxylated AM2201]
Dihydroxylation at pentyl side chain and/or indole moiety [+glucosidation]	[glucosides Mc7, Mc13]	264: dihydroxylated *N*-fluoropentylindole, [392: dihydroxylated AM2201]
Hydroxylation at indole moiety [+glucosidation]	Mc36, [glucoside Mc12]	160: hydroxylated indole, 248: hydroxylated *N*-fluoropentylindole, [376: hydroxylated AM2201]
Hydroxylation at naphthalene moiety [+glucosidation]	Mc40, Mc41, Mc44, Mc45, [glucoside Mc14]	171: hydroxylated naphthalene, [376: hydroxylated AM2201]
Hydroxylation at pentyl side chain	Mc35, Mc37 and Mc39	144: unchanged indole, 248: hydroxylated *N*-fluoropentylindole
Ketone formation at pentyl side chain	Mc43	144: unchanged indole, 246: *N*-fluoropentylindole with ketone
Oxidative defluorination (JWH-018 *N*-(5-hydroxypentyl))	Mc34	144: unchanged indole, 230: hydroxylated *N*-pentylindole
Oxidative defluorination+dihydrodiol formation (JWH-018 dihydrodiol-hydroxy)	Mc4	189: naphthalene with dihydrodiol, 230: hydroxylated *N*-pentylindole
Oxidative defluorination+hydroxylation at indole moiety (JWH-018 dihydroxy)	Mc15, Mc18	160: hydroxylated indole, 246: dihydroxylated *N*-pentylindole
Oxidative defluorination+hydroxylation at naphthalene moiety (JWH-018 dihydroxy)	Mc23, Mc28	144: unchanged indole, 171: hydroxylated naphthalene, 230: hydroxylated *N*-pentylindole
Oxidative defluorination+hydroxylation at pentyl side chain (JWH-018 dihydroxy)	Mc21	144: unchanged indole, 246: dihydroxylated *N*-pentylindole
Oxidative defluorination to carboxylic acid (JWH-018 *N*-pentanoic acid)	Mc33	144: unchanged indole, 244: carboxylated pentylindole
Oxidative defluorination to carboxylic acid+hydroxylation at naphthalene moiety.	Mc20, Mc26	144: unchanged indole, 171: hydroxylated naphthalene, 244: carboxylated pentylindole
Trihydroxylation at indole moiety, naphthalene moiety and pentyl side chain	Mc8, Mc11	160: hydroxylated indole, 171: hydroxylated naphthalene, 264: dihydroxylated *N*-fluoropentylindole
